# IGF-1 decreases portal vein endotoxin via regulating intestinal tight junctions and plays a role in attenuating portal hypertension of cirrhotic rats

**DOI:** 10.1186/s12876-015-0311-5

**Published:** 2015-07-08

**Authors:** Tian-Yu Zhao, Li-Ping Su, Chun-Ye Ma, Xiao-Han Zhai, Zhi-Jun Duan, Ying Zhu, Gang Zhao, Chun-Yan Li, Li-Xia Wang, Dong Yang

**Affiliations:** 1 Department of Gastroenterology, The First Affiliated Hospital of Dalian Medical University, 116000 Dalian, Liaoning province China; 2 Department of Neurology, The Second Affiliated Hospital of Dalian Medical University, 116023 Dalian, Liaoning province China; 3 Department of Clinical Pharmacology, The First Affiliated Hospital of Dalian Medical University, 116000 Dalian, Liaoning province China; 4 Department of Infectious Diseases, The First Affiliated Hospital of Dalian Medical University, 116000 Dalian, Liaoning province China

**Keywords:** Liver cirrhosis, IGF-1, Tight junction, Intestinal barrier, Apoptosis

## Abstract

**Background:**

Intestinal barrier dysfunction is not only the consequence of liver cirrhosis, but also an active participant in the development of liver cirrhosis. Previous studies showed that external administration of insulin-like growth factor 1 (IGF-1) improved intestinal barrier function in liver cirrhosis. However, the mechanism of IGF-1 on intestinal barrier in liver cirrhosis is not fully elucidated. The present study aims to investigate the mechanisms of IGF-1 improving intestinal barrier function via regulating tight junctions in intestines.

**Methods:**

We used carbon tetrachloride induced liver cirrhotic rats to investigate the effect of IGF-1 on intestinal claudin-1 and occludin expressions, serum alanine transaminase (ALT) and aspartate transaminase (AST) levels, severity of liver fibrosis, portal pressures, enterocytic apoptosis and lipopolysaccharides (LPS) levels in portal vein. The changes of IGF-1 in serum during the development of rat liver cirrhosis were also evaluated. Additionally, we assessed the effect of IGF-1 on claudin-1 and occludin expressions, changes of transepithelial electrical resistance (TEER) and apoptosis in Caco-2 cells to confirm in vivo findings.

**Results:**

Serum IGF-1 levels were decreased in the development of rat liver cirrhosis, and external administration of IGF-1 restored serum IGF-1 levels. External administration of IGF-1 reduced serum ALT and AST levels, severity of liver fibrosis, LPS levels in portal vein, enterocytic apoptosis and portal pressure in cirrhotic rats. External administration of IGF-1 increased the expressions of claudin-1 and occludin in enterocytes, and attenuated tight junction dysfunction in intestines of cirrhotic rats. LPS decreased TEER in Caco-2 cell monolayer. LPS also decreased claudin-1 and occludin expressions and increased apoptosis in Caco-2 cells. Furthermore, IGF-1 attenuated the effect of LPS on TEER, claudin-1 expression, occludin expression and apoptosis in Caco-2 cells.

**Conclusions:**

Tight junction dysfunction develops during the development of liver cirrhosis, and endotoxemia will develop subsequently. Correspondingly, increased endotoxin in portal system worsens tight junction dysfunction via decreasing intestinal occludin and claudin-1 expressions and increasing enterocytic apoptosis. Endotoxemia and intestinal barrier dysfunction form a vicious circle. External administration of IGF-1 breaks this vicious circle. Improvement of tight junctions might be one possible mechanism of the restoration of intestinal barrier function mediated by IGF-1.

## Background

Decompensated liver cirrhosis is the end stage of various chronic liver diseases with different etiologies. Complications encompassing variceal bleeding, spontaneous bacterial peritonitis, hepatic encephalopathy, hepatorenal syndrome and hepatic pulmonary syndrome are common presentations of decompensated liver cirrhosis and often life threatening to liver cirrhotic patients. All the complications are the consequences of liver dysfunction and portal hypertension. Intestinal barrier dysfunction is also the consequence of portal hypertension and a risk factor for spontaneous bacterial peritonitis. In recent years, it is found that intestinal barrier interruption is not only the consequence of liver cirrhosis, but also an active participant in the development of liver damage and portal hypertension [1, 2]. Bacterial translocation and increased lipopolysaccharides (LPS) permeability through defective intestinal barrier could cause pro-inflammatory cytokines to be released from polymorphonuclear cells in the portal vein system. Pro-inflammatory cytokines induce nitric oxide synthase (NOS) overexpression and nitric oxide overproduction in the portal vein system, which could cause hyperdynamic circulatory state of the portal vein system, and portal hypertension will ensue subsequently [1, 2]. Moreover, pro-inflammatory cytokines could cause hepatocytic injury and worsen liver dysfunction in liver cirrhosis [3].

Insulin-like growth factor 1 (IGF-1) is a polypeptide with an insulin like structure, and whose receptors are widely distributed in muscles, bones, intestines, testicles and livers [4, 5]. Apart from the pro-growth and trophic effects of IGF-1 on various tissues, IGF-1 could act on intestines to regulate intestinal barrier function [6]. A previous study showed that IGF-1 reduced intestinal barrier permeability through increasing cyclooxygenase-2 (COX-2) expression and decreasing tumor necrosis-alpha (TNF-alpha) expression of intestinal cells [6]. The cytoprotective effect of prostaglandins produced by COX-2 might be one of the mechanisms of IGF-1 improving intestinal barrier function in liver cirrhosis [6]. Some previous studies also indicated that IGF-I upregualted tight junction protein expressions in osteoblast- like MC3T3-E1 cells and reduced apoptosis in mesenchymal stem cells [7, 8]. However, whether IGF-I could regulate enterocytic tight junction proteins and apoptosis to improve intestinal barrier functions is not clear. In the present study, we used carbon tetrachloride (CCL_4_) induced liver cirrhotic rats as the animal models to investigate the effect of IGF-1 on endotoxin levels in portal vein, enterocytic apoptosis and portal pressure. The effect of IGF-1 on claudin-1 and occludin expressions in intestines was also evaluated. We also used Caco-2 cells to confirm the effect of IGF-1 on claudin-1 and occludin expressions in vitro. Transepithelial electrical resistance was employed to evaluate the effect of IGF-1 on cellular monolayer permeability. Furthermore, we also evaluated the effect of IGF-1 on LPS induced apoptosis of enterocytes. Our findings confirmed that IGF-1 was able to decrease portal hypertension by reducing portal vein endotoxin via regulating intestinal tight junction proteins. Our findings also enriched our knowledge about the mechanism of IGF-1 on intestinal barrier and indicated IGF-1 to be a promising therapy for preventing bacterial translocation, reducing hepatocytic damages and attenuating portal hypertension.

## Methods

### Animals

Male Sprague–Dawley rats were obtained from the Animal Center of Dalian Medical University (Dalian, China) and kept under standard laboratory conditions. Rats were acclimatized to the laboratory conditions for one week before further experiments. This study was carried out in strict accordance with the International Council for Laboratory Animal Science. All animal experiments were conducted in accordance with protocols approved by the Experimental Animal Ethical Committee of Dalian Medical University.

### Animal model and experimental design

Thirty male rats (6-week-old) were randomly divided into three groups (ten rats in each group) with the random number. The liver cirrhotic group was injected with CCL_4_ (0.2 ml/kg/d) intraperitoneally for 6 weeks. The control group was administered with sterile normal saline (0.2 ml/kg/d) intraperitoneally for 6 weeks. The IGF-1 group received CCL_4_ (0.2 ml/kg/d) intraperitoneally for 6 weeks and was administered with recombinant human IGF-1 (0.2 μg/kg/d) subcutaneously for 21 days afterwards. All animals were scarified subsequently. Liver, intestines and blood in portal veins and abdominal aortas were harvested for further analyses.

### Cell line and cell culture

The human colorectal adenocarcinoma cell line Caco-2 was purchased from the Chinese Academy of Medical Sciences. Caco-2 cells were cultured in high glucose DMEM (Gibco, Bethesda, MD, USA) supplemented with 10 % fetal bovine serum (Gibco, Bethesda, MD, USA), 1 % nonessential amino acids (Gibco, Bethesda, MD, USA) and 1 % Glutamine (Gibco, Bethesda, MD, USA) at 37 °C with an atmosphere of 5 % CO2.

### Cell experimental design

Caco-2 cells at the logarithmic phase were collected and incubated in a 6-well plate (5 × 10^5^ cells per well) with serum-free DMEM for 24 h. After attachment to the wall, Caco-2 cells were administered with IGF-1 (PeproTech, Rocky Hill, NJ, USA) (100 ng/ml), LPS (Sigma-Aldrich Company, St. Louis, MO, USA) (10 μg/ml), IGF-1 (100 ng/ml) plus LPS (10 μg/ml) and no treatment for 24 h respectively. The final volume of each well was 1 ml and each group was replicated with 6 wells.

### Serum alanine aminotransferase (ALT) and aspartate aminotransferase (AST) analysis

Blood samples without anticoagulant from the abdominal aorta were centrifuged at 3,000 rpm for 15 min to collect the serum. The serum ALT and AST levels were determined with the assay kit (Nanjing Jiancheng Bioengineering Institute, Nanjing, China) according to the manufacturer’s protocols.

### Hemodynamic studies of portal pressure

Rats were anaesthetized with chloral hydrate. Catheters were inserted into the portal vein of rats and the intravascular pressure was detected with the pressure transducers (BL-420 F biological experimental system, Chengdu Technology & Market Co. Ltd., China).

### Plasma endotoxin analysis

Fresh blood was collected from the portal vein of rats and anticoagulated with heparin. Subsequently, the blood was centrifuged at 3,000 rpm for 15 min to collect plasma. Limulus Amebocyte Lysate test kit (Xiamen Huoshiji Cor. Ltd., Xiamen, China) was employed to determine endotoxin concentration according to the manufacturer’s protocols. Briefly, the control standard endotoxin was diluted to 0.01EU/ml, 0.025EU/ml, 0.05EU/ml and 0.1EU/ml in pyrogen free tubes respectively. Afterwards, 100 μl diluted endotoxin was mixed with 100 μl Limulus amoebocyte lysate in the pyrogen free tube and heated at a temperature of 37 °C in a water bath for sixty minutes. The mixed sample was mixed again with 100 μl chromogenic substrate and heated at a temperature of 37 °C in a water bath for another sixty minutes. Azo reagent NO.1, Azo reagent NO.2 and Azo reagent NO.3 were added to the reaction system successively to terminate reactions. Five minutes later, the absorbance at 545 nm of the final mixed sample was measured. The standard curve was depicted with the absorbance as the x-axis and the concentration as the y-axis. Absorbance of the tested plasma endotoxin was measured with the same method and the concentration of plasma endotoxin was calculated from the standard curve. All of the above procedures were conducted in sterile conditions.

### Liver histopathology and evaluation of the severity of liver fibrosis

Fresh liver tissues were fixed in 10 % neutral formalin for 24 h before being embedded in paraffin. After embedded in paraffin, the tissues were cut into sections with a thickness of 5 μm and mounted onto slides. Masson trichrome staining were performed according to standard procedures. Image pro plus software 6.0 was used to evaluate the severity of liver fibrosis. The percentage of fibrosis area in the whole liver area was calculated with five visual fields in one tissue section (×100), and the mean percentage of fibrosis of the five visual fields stood for fibrosis severity.

### Measurement of IGF-1 by Enzyme-linked immunosorbent assay (ELISA)

The levels of serum IGF-1 were measured with the ELISA kits (Beijing Puerweiye Biological Technology Co., Ltd., Beijing, China) according to the manufacture’s protocols. Briefly, the standard IGF-1 solution was diluted to 100 ng/ml, 50 ng/ml, 25 ng/ml, 12.5 ng/ml, 6.25 ng/ml, 3.12 ng/ml and 1.56 ng/ml in tubes respectively. The ELISA method was used to determine the absorbance at 450 nm of each diluted IGF-1 solution. And the standard curve was depicted with the absorbance as the x-axis and the concentration as the y-axis. The absorbance of serum IGF-1 was determined with the same ELISA method and the concentration of serum IGF-1 was calculated from the standard curve.

### Apoptosis assay

Caco-2 cells in monolayer were fixed with 4 % paraformaldehyde, permeabilized with 0.2 % Triton X-100, and blocked with skim milk prior to TUNUEL assay. We used the In situ Cell Death Detection Kit (Roche, Indianapolis, IN, USA) to conduct TUNEL assay according to the manufacturer’s instructions. Fluorescent images were detected with the Leica TCS NT spectral confocal imaging system combined with the Leica DM IRBE inverted microscope (Leica, Solms, Germany).

### Transepithelial Electrical Resistance (TEER) measurement

Caco-2 cells with different treatments were seeded on polycarbonate 12-well Transwell filter inserts (Corning Costar Corp., Cambri dge, MA, USA; pore size 0.4 μm, growth area 1.12 cm^2^) with 2 × 10^5^ cells per well. Before TEER measurement, Caco-2 cells on each well were grown for 21 days and formed a polarized monolayer. The Millicell ERS instrument (Millipore, Bedford, MA, USA) was used to determine the TEER values of the Caco-2 cell monolayer. The changes of TEER were calculated with the formula: TEER = (R1-R0) × A (Ω · cm^2^). R1 and R0 represented the TEER values of the cell monolayer wells and the background wells without cells respectively, and A (cm^2^) represented the surface area of cell monolayer on the insert.

### Western blot analysis

The resected terminal ileum tissues were mixed with the lysis buffer (1 % Triton X-100; 50 mmol/L Tris–HCl, pH 7.6; 150 mmol/L NaCl; and 1 % protease inhibitor) and centrifuged at 12,000 r/min for 5 min at 4 °C. The supernatant was separated and collected. Caco-2 cells were harvested, washed with the cold PBS (PH 7.4) and mixed with the lysis buffer. The total proteins of tissues and Caco-2 cells were exacted with the protein extraction kit (Nanjing KeyGEN Biotech Co. Ltd., Nanjing, China) following the manufacturer’s instructions. The protein concentration was determined with the bicinchoninic acid (BCA) protein assay. After separated with SDS-PAGE, the proteins were transferred to a PVDF membrane. The PVDF membrane was block with 5 % skim milk in Tris-buffered saline containing 0.05 % Tween-20 (TTBS), incubated with the primary antibodies (occludin antibody (Santa Cruz, CA, USA, 1:1000 dilution), claudin-1 antibody (Abcam, Cambridge, MA, USA, 1:1000 dilution) and beta-actin antibody (Zhongshan Goldenbridge Biological Technology, Beijing, China, 1:1000 dilution)) overnight at 4 °C and subsequently incubated with the secondary antibody for 2 h at 37 °C. The bands were photographed and quantified with the ChemiDoc XRS documentation system (Bio-Rad Laboratories). The intensity of the beta-actin band was as the internal reference.

### Reverse transcription-polymerase chain reactions (RT-PCRs) analysis

Total RNA was extracted from the tissues and Caco-2 cells with TRIZOL reagent (Keygen Biotech Co., Ltd, Nanjing, China). We measured the optical density of the products at 260 nm (A260 = 1 for 40 μg/mL RNA) to quantify RNA, and calculated the ratio of the optical density obtained at 260 and 280 nm (pure RNA: A260/A280 = 2.0) to determine the purity of RNA. All the optical analyses were conducted with the UV-1206 spectrophotometer (Shimadzu, Japan). Reverse transcription PCR was performed with a Takara RNA PCR Kit (Takara bio inc., Dalian, China). The primers were listed in Table 1. PCR conditions were as the followings: denaturation at 95 °C for 30 s, followed by 40 cycles of denaturation at 95 °C for 5 s, 30 s at 60 °C for annealing and 30 s at 72 °C for elongation. PCR products were separated by 1.5 % agarose gel electrophoresis subsequently. The product bands were photographed. The density of each band was quantified and adjusted with the internal reference beta-actin.

**Table 1:** The primers of BCL-2, Bax, claudin-1 and occludin of humans and rats

Gene	Forward/Reverse	Sequence 5′-3′
Rat BCL-2	Forward	AAACGTCCAGAGTGCTAC
	Reverse	CAGCCAGATTTAGGTTCA
Human BCL-2	Forward	GAAGCATACCCGTTTAGC
	Reverse	CGAGAACTGGGAGAAGAA
Rat Bax	Forward	GGCGATGAACTGGACAAC
	Reverse	GTGAGTGAGGCAGTGAGGA
Human Bax	Forward	CTGACGGCAACTTCAACTG
	Reverse	GAAAACGCATTATAGACCACA
Rat claudin-1	Forward	GTGGATGTCCTGCGTTTC
	Reverse	GTGTTGGGTAAGAGGTTGTT
Human claudin-1	Forward	CTTCATTCTCGCCTTCCT
	Reverse	TGACAGCCATCCTCATCTT
Rat occludin	Forward	AACCCGAAGAAAGATGGA
	Reverse	TCTGAAGTGATAGGTGGATA
Human occludin	Forward	TATGCCCTCTGCAACCAA
	Reverse	CACCGCTGCTGTAACGAG
Rat beta-actin	Forward	GAGGGAAATCGTGCGTGAC
	Reverse	CTGGAAGGTGGACAGTGAG
Human beta-actin	Forward	GGAGATTACTGCCCTGGCTCCTA
	Reverse	GACTCATCGTACTCCTGCTTGCTG

### Statistical analysis

Data are expressed as mean and standard deviation (SD). One-way ANOVA analysis and least significant difference (LSD) test were employed to determine the difference of means among different groups. *P* <0.05 was considered statistically significant. All statistical analyses were performed with the SPSS16.0 statistical software package (SPSS Inc., Chicago, IL, USA).

## Results

### IGF-1 attenuates liver cirrhosis

**Fig. 1:**
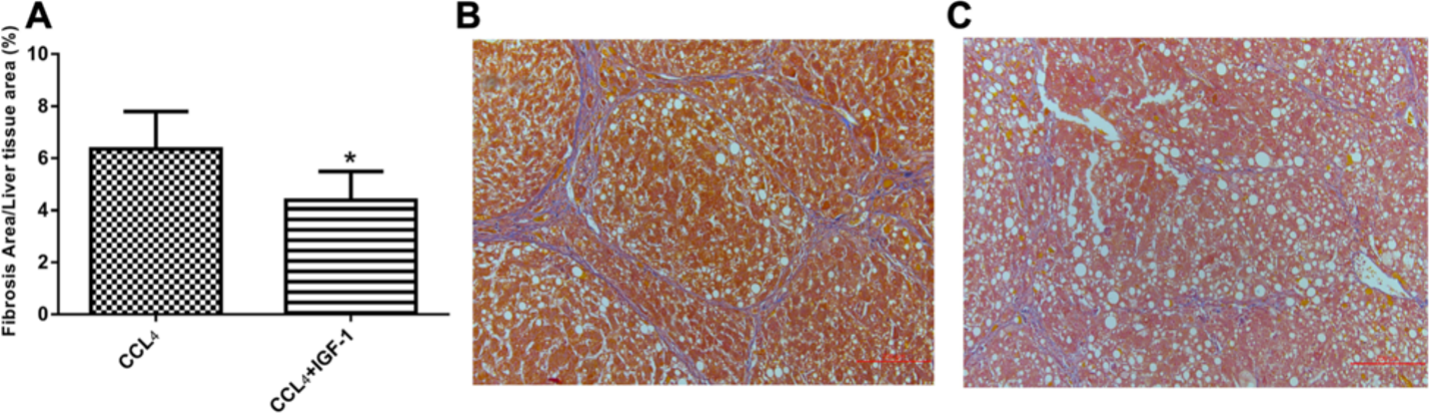
**a** Administration of IGF-1 reduced the severity of fibrosis in liver cirrhosis rats. **b** Representative histology of the cirrhosis without IGF-1 group. **c** Representative histology of the IGF-1 treated cirrhosis group. Data were shown with the mean ± SD. *P <0.01 vs. CCL_4_ group

**Fig. 2:**
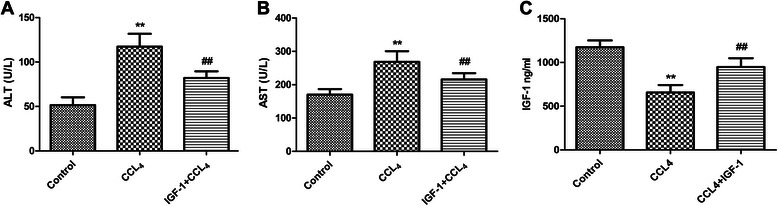
**a** and **b** The levels of serum ALT and AST were increased in liver cirrhosis rats, and administration of IGF-1 reduced the levels of serum ALT and AST in liver cirrhosis rats. **c** IGF-1 was decreased in the development of liver cirrhosis, and administration of IGF-1 restored the levels of IGF-1 in liver cirrhosis rats. Data were shown with the mean ± SD. **P <0.001 vs. control group, ## *P* <0.001 vs. CCL_4_ group

After 6 weeks of CCL_4_ administration, rats developed liver cirrhosis with ascites. Liver cirrhosis was confirmed with liver histology (Fig. 1b and c). Serum ALT and AST levels of liver cirrhosis rats were higher than those of the control rats (Fig. 2a and b), which indicated enhanced hepatocelluar damages in liver cirrhotic rats. Moreover, the IGF-1 level of liver cirrhosis group was reduced compared with that of the control group (Fig. 2c), which indicated IGF-1 insufficiency in liver cirrhosis. External administration of IGF-1 restored IGF-1 level of liver cirrhosis and reduced the levels of serum ALT and AST (Fig. 2a, b and c), which implicated that IGF-1 protected hepatocytes from damaging in liver cirrhosis. Liver fibrosis severity analysis showed that external administration of IGF-1 attenuated liver fibrosis (Fig. 1a, b and c).

### IGF-1 reduces the levels of plasma endotoxin via regulating intestinal tight junctions and subsequently reduces portal hypertension

**Fig. 3:**
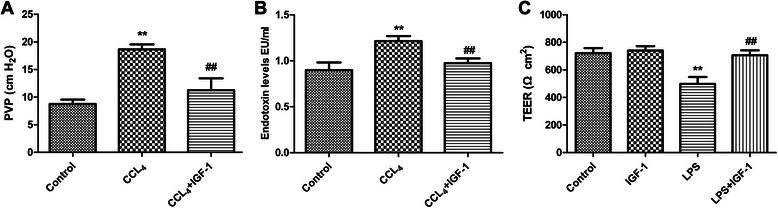
**a** Portal pressure of the liver cirrhosis group was higher than that of the control group significantly, and external administration of IGF-1 reduced portal pressure of the liver cirrhosis. **b** Plasma endotoxin levels of the liver cirrhosis group were higher than those of the control group significantly, and external administration of IGF-1 reduced plasma endotoxin levels of the liver cirrhosis. **c** TEER values of Caco-2 cell monolayer in LPS group were lower than those of Caco-2 cell monolayer in the control group. IGF-1 increased the TEER values of Caco-2 monolayer as compared with those of the LPS group. Data were shown with the mean ± SD. **P <0.001 vs. control group, ## *P* <0.001 vs. CCL_4_ group or LPS group

**Fig. 4:**
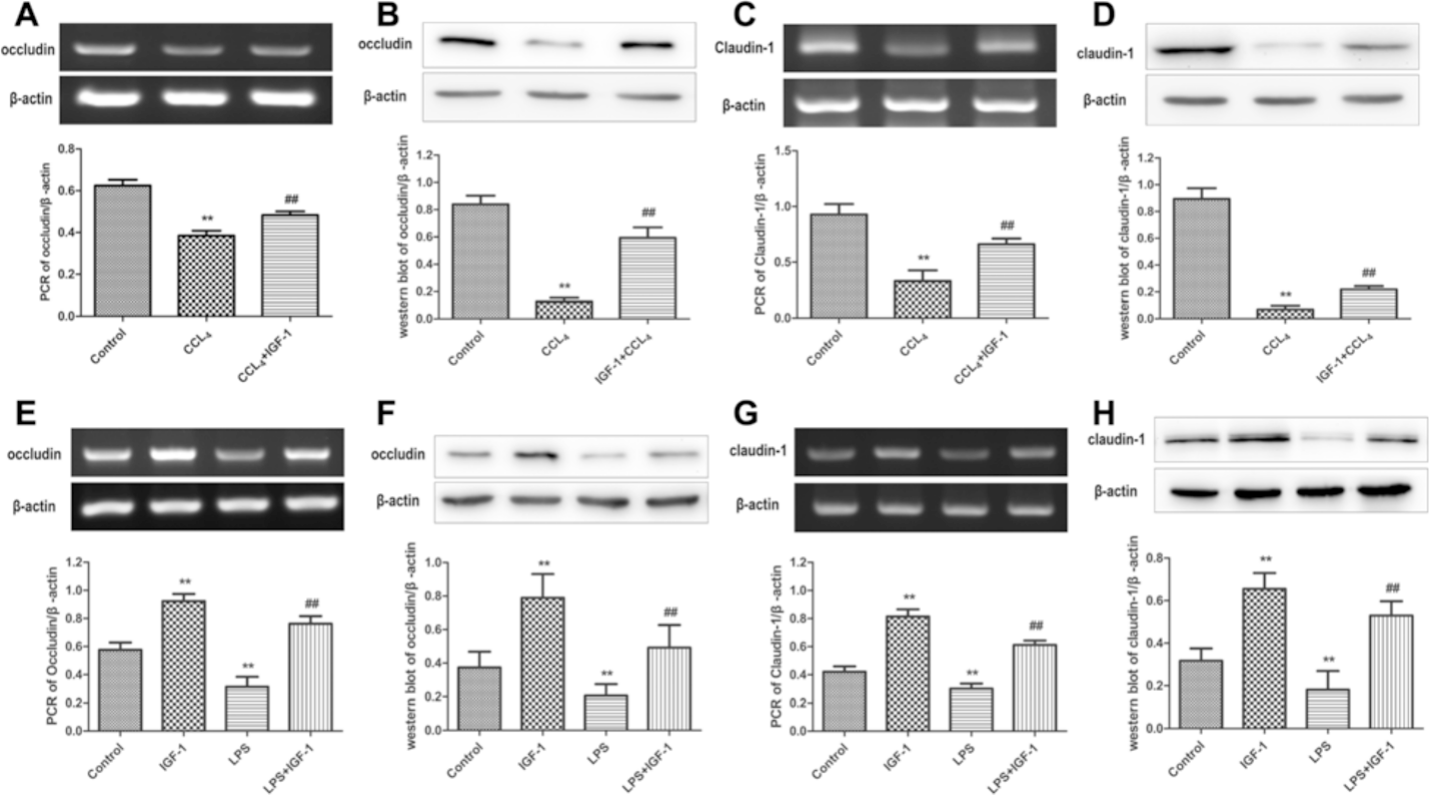
**a** and **b** occludin level in intestines was decreased during the development of liver cirrhosis in rats, and administration of IGF-1 restored occludin level in intestines in liver cirrhosis to some extent. **c** and **d** claudin-1 level in intestines was decreased during the development of liver cirrhosis in rats, and administration of IGF-1 restored claudin-1 level in intestines in liver cirrhosis to some extent. **e** and **f** Administration of LPS to Caco-2 cells reduced occludin level, and external administration of IGF-1 attenuated the effect of LPS on occludin expression in Caco-2 cells. **g** and **h** Administration of LPS to Caco-2 cells reduced claudin-1 level, and external administration of IGF-1 attenuated the effect of LPS on claudin-1 expression in Caco-2 cells. Data were shown with the mean ± SD. **P <0.001 vs. control group, ## *P* <0.001 vs. CCL_4_ group or LPS group

Endotoxin was previously proven to be a precipitating factor in the development of cirrhotic portal hypertension. We postulated that IGF-1 could strengthen intestinal junctions and prevent gut endotoxin from entering portal vein system. Through reducing plasma endotoxin level, IGF-1 could reduce portal hypertension. As expected, portal pressure of the liver cirrhosis group was higher than that of the control group significantly, and external administration of IGF-1 reduced portal pressure of the liver cirrhotic rats (Fig. 3a), which showed that IGF-1 was a negative regulating factor in portal hypertension of liver cirrhosis. Not surprisingly, plasma endotoxin entering portal veins were increased during the development of liver cirrhosis (Fig. 3b), and this effect was via decreased expressions of occludin and claudin-1 in intestines (Fig. 4a, b, c and d),which indicated tight junction dysfunction playing important roles in endotoxemia development of liver cirrhosis. Moreover, external administration of IGF-1 reduced plasma endotoxin levels in portal veins of liver cirrhotic rats (Fig. 3b), and this effect was via increased expressions of occludin and claudin-1 in intestines (Fig. 4a, b, c and d). So IGF-1 improved intestinal tight junction function via upregulating occludin and claudin-1 expressions. With improvement of intestinal tight junction function, endotoxemia would be alleviated. We also used in vitro experiments to confirm our in vivo findings. As expected, LPS reduced the TEER values in Caco-2 cell monolayer (Fig. 3c). Furthermore, we found that IGF-1 increased the TEER values (Fig. 3c) in Caco-2 monolayer through upregulating occludin and caludin-1 expressions (Fig. 4e, f, g and h), which indicated that IGF-1 upregulated these two tight junction proteins in intestinal cells to restore intestinal tight junctions. With this cell experiment, LPS was also proven to be able to cause tight junction dysfunction. Endotoxemia and intestinal barrier dysfunction forms a vicious circle.

### IGF-1 inhibits enterocytic apoptosis in vivo and in vitro

**Fig. 5:**
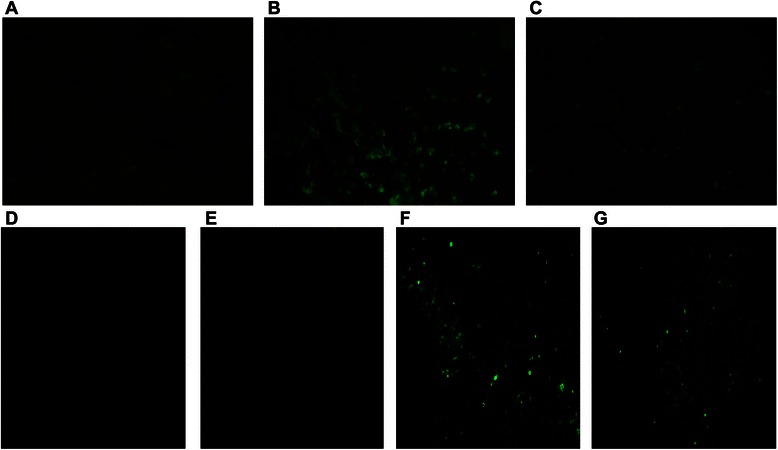
Apoptosis of intestinal cells in rats was increased during the development of liver cirrhosis, and external administration of IGF-1 reduced intestinal cell apoptosis in liver cirrhosis rats: **a** Control group, **b** CCL_4_ group, **c** CCL_4_ + IGF-1 group. LPS induced Caco-2 cell apoptosis and IGF-1 inhibited LPS induced Caco-2 cell apoptosis: **d** Control group, **e** IGF-1 group, **f** LPS group, (G) LPS + IGF-1 group

**Fig. 6:**
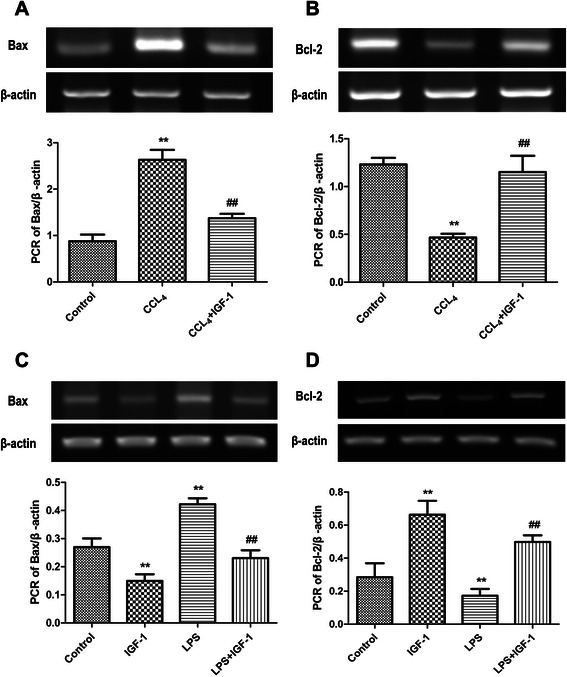
**a** and **b** Bax was increased and Bcl-2 was decreased in intestines during the development of liver cirrhosis in rats, and administration of IGF-1 attenuated Bax and Bcl-2 changes in intestines of liver cirrhosis rats. **c** and **d** Administration of LPS to Caco-2 cells decreased Bax level and increased Bcl-2 level, and IGF-1 attenuated the effect of LPS on Bax and Bcl-2 expressions in Caco-2 cells. Data were shown with the mean ± SD. **P <0.001 vs. control group, ## *P* <0.001 vs. CCL_4_ group or LPS group

We also postulated that high endotoxin levels in portal veins increased enterocytic apoptosis, and increased enterocytic apoptosis caused intestinal tight junction damages subsequently. TUNEL assay was used to evaluate apoptosis in vitro and in vivo. As expected, apoptosis of enterocytes was increased during the development of liver cirrhosis (Fig. 5a and b) via regulating Bax and Bcl-2 (Fig. 6a and b), and external administration of IGF-1 reduced enterocytic apoptosis in liver cirrhotic rats (Fig. 5b and c) through regulating Bax and Bcl-2 (Fig. 6a and b). In vitro experiment with Caco-2 cells proved that LPS promoted apoptosis via Bax and Bcl-2, and IGF-1 attenuated the LPS induced apoptosis through regulating Bax and Bcl-2 (Fig. 6c and d). So IGF-1 improved intestinal barrier function through attenuating apoptosis of enterocytes.

## Discussion

Intestinal barrier dysfunction causing bacterial translocation and endotoxemia is the consequence of decompensated liver cirrhosis. Bacterial translocation is a risk factor for spontaneous bacterial peritonitis, which increases mortality of the liver cirrhosis patients consequently. Moreover, endotoxemia causes pro-inflammatory cytokines to be released from polymorphonuclear cells in the portal vein system. The released pro-inflammatory cytokines worsens portal hypertension and liver damages [3]. Additionally, pro-inflammatory cytokines in portal vein system also attacks intestinal epithelia and aggravates intestinal barrier dysfunction. Therefore, intestinal barrier dysfunction per se is also an active participant in liver cirrhosis not just a passive victim.

Intestinal barrier consists of microorganism barrier, mucus adhering to the apical surface of enterocytes, tight junction complexes sealing adjacent enterocytes, abundant intestinal epithelia, immune barrier (primary immune system and secondary immune system) and the enteric nervous system [9, 10]. Among these intestinal barrier components, abundant intestinal epithelia and tight junctions sealing adjacent intestinal endothelia are the fundamental architectures. Furthermore, abundant intestinal epithelia provide the anchor points to tight junctions and are integral parts of tight junctions. Two epidemiological studies showed intestinal tight junction proteins down-regulated in decompensated liver cirrhosis patients as compared with compensated ones and healthy controls [11, 12]. Additionally, this two excellent case control studies indicated occludin, claudin-1 and claudin-2 as the significant altered tight junction proteins during the liver cirrhosis decompensating process [11, 12]. Our animal experiment indicated that occludin and claudin-1 decreased during liver cirrhosis development, endotoxin in portal system was also found elevated due to the tight junction dysfunction in liver cirrhotic rats. These results are in consistence with the findings of the previous epidemiological studies [11, 12]. Furthermore, we also found that intestinal apoptosis was increased in liver cirrhtic rats. Abundant intestinal epithelia are the fundamental architectures for intestinal barrier and the attachment points of tight junction complexes, increased intestinal apoptosis would cause intestinal tight junction dysfunction in liver cirrhosis subsequently.

Endotoxemia caused by intestinal barrier dysfunction is an active participant in intestinal barrier dysfunction. Previous studies revealed that LPS challenged rats showed increased intestinal permeability and decreased intestinal occludin expression [13, 14]. Our results support endotoxin playing important roles in intestinal tight junction dysfunction. Through cell experiment, we found that LPS reduced the TEER values in Caco-2 cell monolayer. Moreover, the effect of LPS on the permeability of Caco-2 cell monolayer is via down-regulating occludin and claudin-1. Increased LPS permeability through defective intestinal barrier could cause pro-inflammatory cytokines to be released from polymorphonuclear cells in the portal vein system. Pro-inflammatory cytokines induce NOS overexpression and nitric oxide overproduction in the portal vein system, which could cause hyperdynamic circulatory state of the portal vein system and portal hypertension [1, 2, 12]. Additionally, LPS activates Kupffer cells secreting vasoconstrictors such as thromboxane A2 and cysteinyl leukotrienes, which increase intrahepatic resistance and promote portal hypertension development [15, 16]. Taken together, endotoxemia and intestinal barrier dysfunction (especially intestinal tight junction dysfunction) very likely form a vicious circle [11], breaking this vicious circle may have great clinical significance to liver cirrhosis patients.

IGF-I is a polypeptide with an insulin like structure, whose receptor is also distributed in intestines [4, 5]. IGF-1 is reduced in liver cirrhosis patients, and negatively correlated with MELD score and liver cirrhosis severity [17–19]. As liver is the major synthesis site for IGF-1 and growth hormone (GH) receptor, reduced IGF-1 in liver cirrhosis could be explained by reduced liver function for synthesizing IGF-1 and impaired GH response in liver [19–23]. Moreover, LPS penetrating defect intestinal barrier in liver cirrhosis could also reduce GH releasing in pituitory, IGF-1 synthesis and release in liver would be reduced consequently [24–26]. IGF-I has trophic effects on intestinal epithelia, so IGF-1 deficiency could cause intestinal barrier dysfunction. A previous study showed that IGF-1 reduced intestinal barrier permeability through increasing COX-2 expression and decreasing TNF-alpha expression of enterocytes [6]. The cytoprotective effect of prostaglandin produced by COX-2 might be one of the mechanisms of IGF-1 improving intestinal barrier function in liver cirrhosis [6]. However, whether IGF-I could regulate tight junction to improve intestinal barrier is not clear. So we evaluated the effect of IGF-1 on tight junction in vivo and in vitro. Our animal experiment showed that external administration of IGF-1 reduced endotoxemia in liver cirrhotic rats via up-regulating occludin and claudin-1 expression in intestines. Additionally, external administration of IGF-1 also attenuated enterocytic apoptosis, which ensured abundant tight junction attaching points. Through these mechanisms, intestinal tight junction function would be improved. Cell experiment confirmed our findings in animal experiment. As shown above in the results section, IGF-1 countered the effect of LPS on intestinal tight junction protein expression and apoptosis. IGF-1 is able to break the vicious circle of endotoxemia and intestinal barrier dysfunction.

Previous studies also indicated IGF-1 having the ability of anti-fibrosis. Gene transfer of IGF-1 to the hepatic tissues is proven to be able to improve liver histology and functions in established liver cirrhotic rats [27]. Our data confirm that external administration of IGF-1 can reduce hepatocellular injury and attenuate liver cirrhosis, which is consistent with that of the previous studies [28, 29]. Our explanation is that IGF-1 reduces portal LPS level through improving intestinal barrier function, reduced LPS alleviates hepatic stellate cells (HSC) activation which is a key player in the development of liver cirrhosis [30, 31]. The trophic effect of IGF-1 on hepatocytes also possibly contributes to the improvement of liver cirrhosis. However, the panorama of the mechanism of IGF-1 alleviating liver fibrosis still needs to be elucidated.

## Conclusions

In summary, tight junction dysfunction develops during the development of liver cirrhosis, and endotoxemia will ensue subsequently. Correspondingly, increased endotoxin in portal system worsens tight junction dysfunction via decreasing intestinal occludin and claudin-1 expressions and increasing enterocytic apoptosis. Endotoxemia and intestinal barrier dysfunction form a vicious circle. External administration of IGF-1 breaks this vicious circle. Improvement of tight junctions might be one possible mechanism of the restoration of intestinal barrier function mediated by IGF-1. Moreover, improved intestinal barrier by IGF-1 reduces portal LPS and attenuates Kuppfer cell and HSC activations. Decreased inflammation, reduced HSC activation and the trophic effect of IGF-1 on hepatocytes attenuates liver cirrhosis. Our data further supports IGF-1 as a potential therapy for liver cirrhosis and enriches our knowledge about IGF-1 on intestinal barrier dysfunction.

## Abbreviations

IGF-1: Insulin-like growth factor 1
ALT: Alanine transaminase
AST: Aspartate transaminase
LPS: Lipopolysaccharides
TEER: Transepithelial electrical resistance
NOS: Nitric oxide synthase
COX-2: Cyclooxygenase-2
TNF-alpha: Tumor necrosis-alpha
CCL_4_: Carbon tetrachloride
